# Increased IL-2 and Reduced TGF-β Upon T-Cell Stimulation are Associated with GM-CSF Upregulation in Multiple Immune Cell Types in Multiple Sclerosis

**DOI:** 10.3390/biomedicines8070226

**Published:** 2020-07-18

**Authors:** Jehan Aram, Nanci Frakich, Elena Morandi, Mohammed Alrouji, Amal Samaraweera, David Onion, Ian Spendlove, Sergio L. Colombo, Radu Tanasescu, Bruno Gran, Cris S. Constantinescu

**Affiliations:** 1 Division of Clinical Neuroscience, Section of Clinical Neurology, University of Nottingham, Nottingham NG7 2UH, UK; jehan_ph_tarzean@yahoo.com (J.A.); nanci.frakich@nottingham.ac.uk (N.F.); elena.morandi@inserm.fr (E.M.); malrouji@gmail.com (M.A.); amal.samaraweera@doctors.org.uk (A.S.); r.tanasescu@nottingham.ac.uk (R.T.); bruno.gran@nuh.nhs.uk (B.G.); 2 Inserm, Centre de Physiopathologie de Toulouse Purpan (CPTP), 31300 Toulouse, France; 3 College of Applied Medical Sciences, Shaqra University, Shaqra 11961, Saudi Arabia; 4 Department of Neurology, Nottingham University Hospitals NHS Trust, Nottingham NG7 2UH, UK; 5 Royal Victoria Infirmary, Newcastle upon Tyne NHS Foundation Trust, Newcastle NE1 4LP, UK; 6 Ex vivo Cancer Pharmacology Centre of Excellence, University of Nottingham, Nottingham NG7 2UH, UK; David.onion@nottingham.ac.uk; 7 Academic Clinical Oncology, University of Nottingham, Nottingham NG7 2UH, UK; ian.spendlove@nottingham.ac.uk; 8 Centre for Diabetes, Chronic Diseases, and Ageing, School of Science and Technology, Nottingham Trent University, Nottingham NG11 8NS, UK; Sergio.colombo@ntu.ac.uk; 9 Department of Neurology, Colentina Hospital, University of Medicine and Pharmacy Carol Davila, Bucharest 021172, Romania

**Keywords:** GM-CSF, human, MS/EAE, PBMC, T cells, B cells, NK cells, monocytes

## Abstract

Granulocyte macrophage colony stimulating factor (GM-CSF) is a pro-inflammatory cytokine produced by immune cells. Recent evidence suggests that GM-CSF plays an important role in multiple sclerosis (MS) pathogenesis. We investigated the expression and regulation of GM-CSF in different immune cells in MS. We also investigated the differentiation and frequency of GM-CSF-producing Th cells that do not co-express interferon (IFN)-γ or interleukin-17 (IL-17) (Th-GM cells) in MS. We found a significant increase in the percentage of GM-CSF-expressing Th cells, Th1 cells, Th-GM cells, cytotoxic T (Tc) cells, monocytes, natural killer (NK) cells, and B cells in PBMC from MS patients stimulated with T cell stimuli. Stimulated PBMC culture supernatants from MS patients contained significantly higher levels of IL-2, IL-12, IL-1β, and GM-CSF and significantly lower levels of transforming growth factor (TGF-)β. Blocking IL-2 reduced the frequency of Th-GM cells in PBMC from MS patients. The frequency of Th-GM cells differentiated in vitro from naïve CD4^+^ T cells was significantly higher in MS patients and was further increased in MS with IL-2 stimulation. These findings suggest that all main immune cell subsets produce more GM-CSF in MS after in vitro stimulation, which is associated with defective TGF-β and increased IL-2 and IL-12 production. Th-GM cells are increased in MS. GM-CSF may be a potential therapeutic target in MS.

## 1. Introduction

Multiple sclerosis (MS) is an immune-mediated inflammatory disease of the central nervous system (CNS) characterised by inflammation, demyelination, axonal damage, and gliosis [1]. The exact underlying causes of MS are still unknown [2,3]. In MS, there is an imbalance between pro-inflammatory and the anti-inflammatory factors, favouring the former. Granulocyte macrophage colony stimulating factor (GM-CSF) is a pro-inflammatory cytokine that acts on mature myeloid cells to activate them [4,5,6]. It is also a haematopoietic growth factor that stimulates the formation of granulocytes and macrophages (mature myeloid cells) from precursor cells in the bone marrow [7]. It is expressed by many cell types, including haematopoietic lineage cells. GM-CSF receptor (GMR) is expressed by many cell types, including monocytes, macrophages, antigen presenting cells (APCs), neurons, astrocytes, and oligodendrocytes. This suggests that GM-CSF is involved in physiological regulation of these cells [8].

The important role of GM-CSF in inflammatory demyelination has been demonstrated in studies of the experimental autoimmune encephalomyelitis (EAE) model of MS. Th cells (whether Th1 or Th17) lacking GM-CSF are unable to transfer EAE, GM-CSF-deficient mice are resistant to EAE induction, and GM-CSF antibodies prevent the onset of EAE [9,10,11,12,13]. Moreover, overexpression of GM-CSF leads to more severe EAE by increasing the influx of inflammatory cells into the CNS [14]. GM-CSF was also found to enhance long-term disability in EAE [15]. In addition, myelin peptide-reactive Th1 and Th17 cells that lack GM-CSF failed to transfer EAE [11,12]. A recent study has shown a distinct pathogenic GM-CSF-producing Th cells in EAE [16]. Another study found a distinct Th subtype that mainly express GM-CSF and IL-3 and is essential for EAE pathogenesis [17]. In MS, the role of GM-CSF has also been demonstrated, and the evidence is strengthened by recent studies. GM-CSF levels in the cerebrospinal fluid (CSF) were shown to be elevated in the active stage of MS [18]. Recent studies have found that GM-CSF is highly expressed by CD4 and CD8 T cells in the brains of MS patients [19] and that GM-CSF-producing CD4^+^ T cells are increased in the cerebrospinal fluid (CSF) of MS patients [20]. The GM-CSF receptor is highly expressed in MS lesions [21]. Our collaborators and we identified the role of MS-associated IL-2R polymorphism in the induction by IL-2 of GM-CSF in Th cells [22]. Another recent study has shown a distinct GM-CSF-only producing the Th cell subtype (ThGM) in the CSF of MS patients [23]. Importantly, a seminal study shows that such GM-CSFs predominantly producing Th cells are CXCR4^+^, have pathogenic features in MS, and expand the spectrum of immune cells overexpressing GM-CSF in MS beyond T and B cells [24]. The mechanisms of induction and in particular the deficit of regulatory factors that allow GM-CSF upregulation in MS have not been thoroughly studied.

In this study, we investigated GM-CSF expression and regulation in multiple stimulated immune cell subsets in the peripheral blood of people with MS. We show that the percentage of GM-CSF-expressing stimulated peripheral blood mononuclear cells (PBMC) subsets (Th cells, Tc cells, monocytes, natural killer (NK) cells, and B cells) is higher in MS patients when compared to healthy controls (HCs). The frequency of non-Th1 non-Th17 GM-CSF-producing Th cells (Th-GM cells) and of Th1 cells expressing GM-CSF is higher in MS patients. Based on the percentage of each immune cell subset expressing GM-CSF, Th and Tc cells are the main GM-CSF producers among the immune cell subsets tested. Blocking IL-2 and IL-12 significantly reduces GM-CSF expression by Tc, NK, and B cells in MS patients but not in these cell types for HCs, implicating IL-2 and IL-12 as important stimulatory pathways for GM-CSF in MS. Unlike the study by Galli et al. [24] we stimulate mixed PBMC populations with T cell-specific stimuli to determine if these stimuli affect GM-CSF expression in T and non-T cells and, thus, indicate a T cell-derived effect in non-T cells.

## 2. Subjects and Methods

### 2.1. Subjects

All sample collection and experimental work were approved by the Nottingham Research Ethics Committee 2 (08/H0408/167; approved 05/01/2009), and the study was conducted in accordance with the guidelines of the World Medical Association’s Declaration of Helsinki (most recent revision). All participants (healthy controls (HCs) and MS patients) signed an informed consent before participation. All participating patients had untreated relapsing MS (relapsing-remitting MS (RRMS) and secondary progressive MS (SPMS) in relapsing stage; RRMS, *n* = 38; SPMS *n* = 9). Patients were ≥18 years old, had Expanded Disability Status Scale (EDSS) scores ≤ 6.5, and were relapse free for at least 1 month before recruitment. Exclusion criteria were being pregnant or breast-feeding, having serious infections or other conditions (hepatic, renal, psychiatric, addiction, pulmonary, cardiac, or malignancy), having had a vaccination within 6 months of blood collection, having treatment with immuno-modulatory or immunosuppressive therapies within 1–12 months (depending on the type of therapy) of recruitment, or having a coexistent disease that needs to be treated with such medications. Some of the patients recruited were previously treated with interferon (IFN)-β, daclizumab, copaxone, or fingolimod and had discontinued immunomodulatory therapy for ≥2 months before participation mainly in anticipation of treatment switch. In the patients recruited, there was a gap of a minimum of 3 months between last clinical relapse and time of participation.

### 2.2. Cell Culture and Stimulation

PBMCs were isolated by standard density gradient centrifugation protocol using Histopaque-1077 (Sigma-Aldrich, St. Louis, MO, USA). Fresh or thawed PBMC (1 × 10^6^ cells/well) were cultured in 24-well plates with RPMI medium containing 10% fetal calf serum (FCS), 100 units/mL of penicillin, 0.1 mg/mL of streptomycin, and 2 mM of glutamine (all from Sigma-Aldrich). Cells were either left unstimulated or stimulated with soluble anti-CD3 and anti-CD28 antibodies (1 μg/mL each; BD Biosciences, Franklin Lakes, NJ, USA) and incubated for 5 days in a 37 °C incubator with humidified atmosphere and 5% CO_2_. Individual experiments did not mix fresh and frozen cells.

For cytokine blocking, cells were treated with one or more of the following human antibodies or antagonists (all from R&D Systems) to reach a final concentration of 10 μg/mL each: anti-IL-2 and anti-IL-2R-alpha, anti-IL-12p70, anti-IL-12/23p40, anti-IL-1β and recombinant human IL-1RA, and mouse IgG1 isotype control.

### 2.3. NK Cell Isolation and Stimulation

After PBMC isolation, NK cells were magnetically isolated using an NK isolation kit (Miltenyi Biotec, Bergisch Gladbach, Germany) via negative selection following the manufacturer’s instructions. NK cells were counted and checked for purity (CD3^-^ CD56^+^ ≥ 90%). They were resuspended in RPMI medium with 15% FCS, 100 units/mL of penicillin, 0.1 mg/mL of streptomycin, and 2 mM of glutamine and distributed in a 24-well plate (1 × 10^5^ cells/well). NK cells were either left unstimulated or stimulated with one of the following: rhIL-15 (100 ng/mL) (R&D Systems) + rhIL-1β (10 ng/mL) (Peprotech, Cranbury, NJ, USA), rhIL-15 (100 ng/mL) + rhIL-18 (100 ng/mL) (R&D Systems), and rhIL-2 (10 ng/mL) + rhIL-12 (10 ng/mL) (Peprotech). Cells were incubated for 3 days at 37 °C with 5% CO_2_.

### 2.4. Naïve CD4 T Cell Isolation and Stimulation for Identification of Th-GM Cells

After PBMC isolation, naïve CD4 T cells were isolated using magnetic Naïve CD4^+^ T Cell Isolation Kit II (Miltenyi Biotec) via negative selection, following the manufacturer’s instructions. They were counted and checked for purity (≥90% CD4^+^ CD45RA^+^). Naïve CD4 T cells were distributed in a 24-well plate and divided into five wells (1 × 10^6^ cells/well) left either unstimulated or stimulated with soluble anti-CD3 (3 μg/mL) and anti-CD28 (1 μg/mL, both from BD Biosciences). Stimulated cells were treated with or without any of the following: rhIL-2 (50 ng/mL), rhIL-7 (20 ng/mL), or their combination (both from Peprotech). Cells were incubated for 7 days at 37 °C with 5% CO_2_.

### 2.5. Cell Identification and Analysis by Flow Cytometry

Before staining protocols, cells were restimulated for the last 5 h with phorbol myristate acetate (PMA, 50 ng/mL, Sigma) and ionomycin (I, 0.5 μg/mL, Sigma) in the presence of brefeldin A (10 μg/mL, Sigma). Cultured cell suspensions were placed in fluorescence activated cell sorter (FACS) tubes, including the negative control, single colour controls, and fluorescence minus one (FMO) controls. They were centrifuged (300 × RCF for 5 min), washed once with phosphate-buffered saline (PBS) (1 mL, Sigma), and then resuspended in 1 mL PBS. They were then stained with dead cell stain (near IR 1:1000, Invitrogen, Waltham, MA, USA). Cells were left for 20 min at room temperature in the dark, washed once in FACS buffer (PBS + 2% FCS), and fixed by adding 0.25 mL of Fix/Perm solution (BD Biosciences) for 20 min, keeping them at 4 °C. After fixation, cells were washed twice with perm/wash solution 1× (BD Biosciences). Surface and intracellular staining were performed afterwards using the following antihuman antibodies: CD3-ECD (UCHT1, Beckman Coulter, Brea, CA, USA), CD8-PeCy-7 (SFCI21Thy2D3, Beckman Coulter), CD8-BV785 (RPA-T8, BioLegend, San Diego, CA, USA), CD56-PE (HCD56, BioLegend), CD56-BV605 (NCAM16.2, BD Biosciences), CD14-FITC (HCD14, BioLegend), CD20-PeCy7 (2H7, BioLegend), CD116-APC (REA211, Miltenyi Biotec), GM-CSF-Efluor^®^ 660 (GM2F3, eBioscience, Waltham, MA, USA), GM-CSF-BV421 (BVD2-21C11, BD Biosciences), IL-3-APC (BVD3-1F9, Miltenyi Biotec), IL-17-PE (EBio64CAP17, eBioscience), and IFN-γ-FITC (45.15, Beckman Coulter). Cells were incubated for 30 min at 4 °C, then washed once with perm/wash 1× (1 mL) and once with FACS buffer (1 mL), and finally resuspended in 0.3 mL fixation buffer (BD Biosciences). Cells were analysed on Beckman Coulter Mo-Flo XDP. Typically, a pilot experiment was run on 2–3 samples each from MS patients and controls (where more aliquots were available), validated in a second set of samples of similar size, and then repeated in all samples tested at the same time and, where possible, with duplicate or triplicate measurements per sample.

### 2.6. Cytokine Determination by Multiplex Bead Assay and ELISA

After fresh PBMC isolation and stimulation for 5 days with anti-CD3/anti-CD28, 0.5 mL culture supernatants were taken from unstimulated and stimulated wells, centrifuged, and stored at −20 °C until they were used in multiplex bead assay. The Milliplex^®^ MAP kit (EMD Millipore, Burlington, MA, USA) was used to detect GM-CSF, IFN-γ, IL-2, IL-7, IL-10, IL-12p70, IL-17, and IL-23. The ProcartaPlex^®^ (Affymetrix eBioscience) kit was used to detect IL-1β, IL-6, IL-15, and IL-18. An ELISA kit (BioLegend) was used to detect free active TGF-β1. Samples were processed according to manufacturer’s protocols. Multiplex samples were run on a Bio-Rad (Bio-Plex^®^ 200 System) machine. ELISA samples were run on Benchmark plus microplate reader (Bio-Rad).

### 2.7. Statistics and Data Analysis

Flow cytometry data were analysed using Kaluza 1.5 software (Beckman Coulter). For simplicity, PBMC subtypes were named and analysed based on their canonical function or, for Th-GM, their non-expression of signature Th1 and Th17 cytokines, i.e., helper T cells (Th) = CD4^+^ cells; Th1 = CD4^+^ IFN-γ^+^ cells; Th17 = CD4^+^ IL-17^+^ cells; Th-GM = CD4^+^IL-17^-^IFN-γ^−^ cells; cytotoxic T cells (Tc) = CD3^+^ CD8^+^ CD56^-^ cells; NK cells = CD3^-^ CD56^+^ cells; B cells = CD3^-^ CD20^+^ cells; and monocytes = CD3^-^ CD14^+^ cells. Instead of direct staining of cells with CD4^+^, Th cells were identified as CD3^+^ CD8^-^because of downregulation of CD4 upon stimulation [25,26]. Multiplex bead assay data were analysed using Bio-Plex Manager Software. Data were combined, and statistical analysis was performed using GraphPad Prism 6 (GraphPad Software, La Jolla, CA, USA). The type of statistical test applied depends on whether data were normally distributed (measured with D’Agostino and Pearson normality test), and the number of groups was measured, which is specified in each result section.

## 3. Results

### 3.1. Demographic and Clinical Characteristics of Recruited Patients and Control Subjects

In total, samples were obtained from 47 patients with relapsing MS (38 relapsing remitting, RR, 9 secondary progressive, SP MS): 35 females and 12 males. The mean (SD) age was 43 (12) with a range of 21–67. The mean (SD) EDSS score was 3.3 (1.8), range 1–6.5. The mean (SD) duration of the disease, counting from the onset of the first symptoms, was 7.6 (5,2), range 9 months–27 years. Clinical and demographic characteristics of the patients are provided in Table S1. A total of 10 healthy controls, 8 females and 2 males, contributed samples for this project. Their mean (SD) age was 43 (13) with a range of 24–58.

### 3.2. Higher Frequency of GM-CSF-Expressing Th1 Cells in PBMC of MS Patients after In Vitro Stimulation

**Figure 1:**
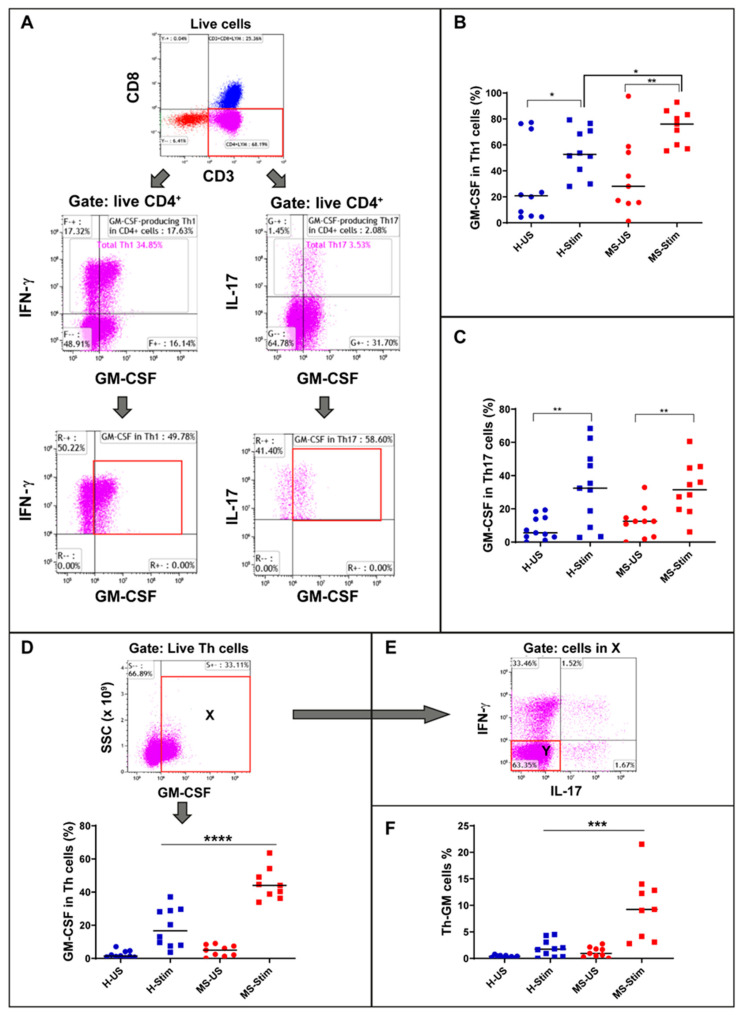
Granulocyte macrophage colony stimulating factor (GM-CSF) expression in Th1, Th17, Th, and Th-GM cells: Frozen isolated peripheral blood mononuclear cells (PBMC) were thawed and stimulated with anti-CD3/anti-CD28 for 5 days and restimulated with phorbol myristate acetate (PMA)/ionomycin (I) in the presence of brefeldin A for the last 5 h. (**A**) Live CD4^+^ cells were gated for Th1 (middle left panel) and Th17 expression (middle right panel). Then, the corresponding percentage of GM-CSF-producing cells were evaluated (bottom panels). (**B**) Global analysis of Th1 GM-CSF-producing cells, both in unstimulated and stimulated healthy control and multiple sclerosis (MS) samples: The paired unstimuated/stimulated (US/S)data for each sample are shown in Figure S1. (**C**) Global analysis of Th17 GM-CSF-producing cells, both in unstimulated and stimulated HC and MS samples. (**D**) Live CD4^+^ (Th) were gated for GM-CSF expression (top panel, population X) and analysed both in unstimulated and stimulated HC and MS samples (lower panel). (**E**) Population X gated cells were used to identify double negatives for IL17 and IFN-γ (population Y). (**F**) Global analysis of non-Th1, non-Th17 Th cells expressing GM-CSF (Th-GM), both in unstimulated and stimulated HC and MS samples, was done using the equation Th-GM (%) = X × Y × 100% (where X and Y are expressed as decimal values). H-US: unstimulated cells from healthy controls; H-Stim: stimulated cells from healthy controls; MS-US: unstimulated cells from MS patients; MS-Stim: stimulated cells from MS patients; and SSC: side scattered. Horizontal lines are medians. * *p* < 0.05; ** *p* < 0.01; *** *p* < 0.001; and **** *p* < 0.0001.

Using a flow cytometry strategy (Figure 1A), we first examined GM-CSF expression in the most frequently represented subset of Th cells in the PBMC: those expressing IFN-γ (Th1 cells, Figure 1A bottom left). We found that the GM-CSF expression was induced significantly in both groups. Moreover, the percentage of GM-CSF-expressing Th1 cells (phenotyped as CD3^+^ CD8^-^ IFN-γ^+^ GM-CSF^+^ cells) out of the total Th1 cells (phenotyped as CD3^+^ CD8^-^ IFN-γ^+^ cells) was higher in in vitro stimulated PBMS from MS patients when compared to those from HCs (HCs *n* 10, MS *n* = 9, *p* = 0.024 unpaired *t*-test, Figure 1B, Figure S1). We also compared the expression of GM-CSF in cells co-expressing IL-17 (Th17 cells, Figure 1A bottom right). We found that the GM-CSF expression was induced significantly in both groups; however, no significant differences in GM-CSF expressing Th17 cells (phenotyped as CD3^+^ CD8^-^ IL-17^+^ GM-CSF^+^ cells) between MS patients and controls were observed (*p* = 0.94) (Figure 1C). The frequencies of GM-CSF^-^ Th1 or Th17 cells after in vitro stimulation were no different between MS patients and controls (data not shown). Details of cell gating and setting quadrants strategy for the flow cytometry analysis are shown in Figures S2 and S3.

### 3.3. Higher Percentage of Non-Th1 Non-Th17 Th Cells Expressing GM-CSF (Th-GM Cells) in PBMC of MS Patients after In Vitro Stimulation

We observed the presence of Th cells that expressed GM-CSF but not IFN-γ or IL-17 in both HCs and MS patients (Figure 1E). We examined the frequency of these cells (CD3^+^ CD8^-^ IFN-γ^−^ IL-17^-^ GM-CSF^+^) among the overall Th cells and found a statistically significant increase in these cells in PBMC from MS patients after in vitro stimulation when compared to those from HCs (HCs *n* = 10, MS patients *n* = 9, *p* = 0.0009 unpaired t-test, Figure 1F). This finding indicates that this novel set of Th cells (Th-GM) is upregulated in MS upon stimulation.

### 3.4. The Frequency of GM-CSF-Expressing Immune Cells Types after In Vitro Stimulation of PBMC Is Higher in MS

**Figure 2:**
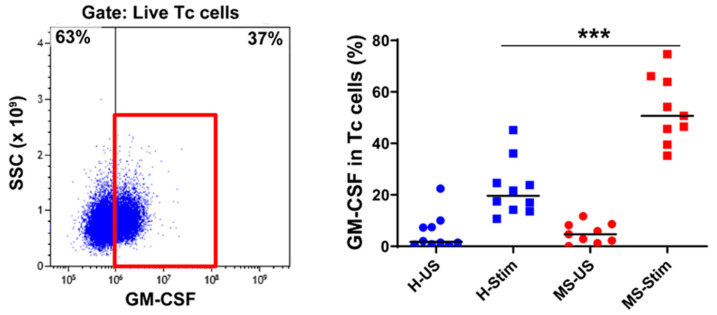
GM-CSF expression in cytotoxic T (Tc) cells: Fresh isolated PBMC were stimulated with anti-CD3/anti-CD28 for 5 days and restimulated with PMA/I in the presence of brefeldin A during the last 5 h. The left panel shows a representative flow cytometry plot analysis in which cells were gated for live Tc cells (full details on the gating strategy can be found in Figure S2). Then, the percentage of GM-CSF-producing cells was counted, and the collective results in healthy controls (H) and MS patients are shown (right panel). US: unstimulated cells; Stim: stimulated. Horizontal lines are medians. *** *p* < 0.001.

**Figure 3:**
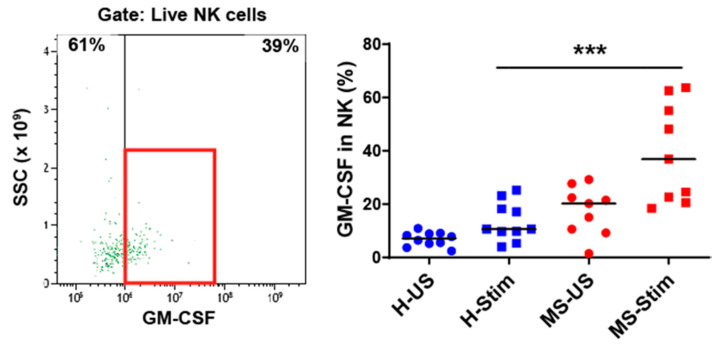
GM-CSF expression in natural killer (NK) cells: Fresh isolated PBMC were stimulated with anti-CD3/anti-CD28 for 5 days and restimulated with PMA/I in the presence of brefeldin A during the last 5 h. The left panel shows a representative flow cytometry analysis in which cells were gated for live NK cells (full details on the gating strategy can be found in Figure S2). Then, the percentage of GM-CSF-producing cells was counted, and the collective results in healthy controls (H) and MS patients are shown (right panel). US: unstimulated cells; Stim: stimulated cells. Horizontal lines are medians. *** *p* < 0.001.

**Figure 4:**
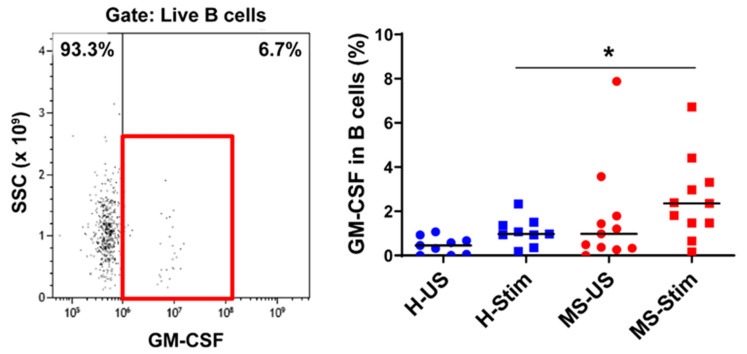
GM-CSF expression in B cells: Frozen isolated PBMC were thawed and stimulated with anti-CD3/anti-CD28 for 5 days and restimulated with PMA/I in the presence of brefeldin A during the last 5 h. The left panel shows a representative flow cytometry analysis in which cells were gated for live B cells (full details on gating in Figure S2). Then, the percentage of GM-CSF-producing cells was counted, and the collective results in healthy controls (H) and MS patients are shown (right panel). US: unstimulated cells; Stim: stimulated cells. Horizontal lines are medians. * *p* < 0.05.

**Figure 5:**
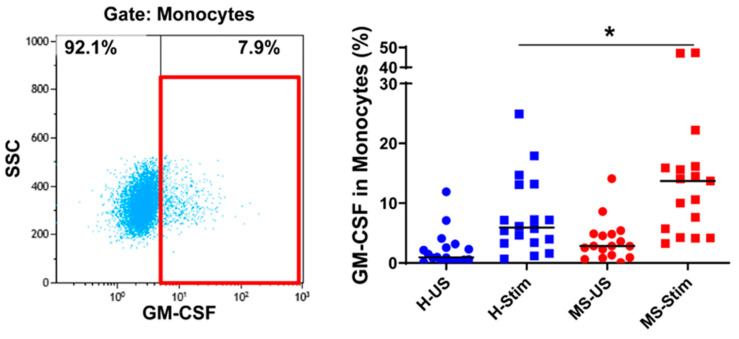
GM-CSF expression in monocytes: Fresh isolated PBMC were stimulated with phorbol dibutyrate (PDB) and ionomycin in the presence of brefeldin A for 16 h. The left panel shows a representative flow cytometry analysis in which cells were gated for monocytes (full details on gating in Figure S4). Then, the percentage of GM-CSF-producing cells was counted, and the collective results in healthy controls (H) and MS patients are shown (right panel). US: unstimulated cells; Stim: stimulated cells. Horizontal lines are medians. * *p* < 0.05.

We also examined the expression of GM-CSF in overall Th cells (Figure 1D), Tc (CD8^+^) cells (Figure 2), NK cells (Figure 3), B cells (Figure 4), and monocytes (Figure 5) in the mixed PBMC. After culturing and stimulating PBMC as above, we found a higher frequency of all these cell types expressing GM-CSF in the PBMC from MS patients compared to controls. This shows increased stimulated production of GM-CSF across multiple immune cell lineages in MS. The cells expressing the highest proportion of GM-CSF were Th cells, Tc cells, and monocytes. Although NK cells represented a small proportion of PBMC, a significant proportion expressed GM-CSF (Figure 3). It should be noted that PMA/ionomycin stimulation downregulates CD56 expression [27]; therefore, not all NK cells were detected in this experiment. We also tried to examine natural killer T (NKT) cells, but very few cells were seen in flow cytometry, which did not allow analysis. NK cells and their cytokine expression in MS have not been extensively studied. Our finding of increased GM-CSF expression in NK cells in PBMC of MS patients stimulated with anti-CD3/anti-CD28, a T cell stimulus, suggested that T cell stimulation upregulates NK cell GM-CSF. We proceeded to determine whether GM-CSF expression is also increased in isolated purified populations of NK cells from people with MS. We chose the stimuli and combination thereof that are known or suspected to be optimal inducers of GM-CSF in NK cells [28]. Isolated and stimulated NK cells (with IL-15 + IL-1β, IL-15 + IL-18, or IL-2 + IL-12) did not show any differences in their GM-CSF expression between HCs and MS patients (Figure S5). There was also no difference in the expression of IFN-γ between control and MS patient (results not shown) expression. This indicates that the increased GM-CSF in NK cells from MS patients after anti-CD3/anti-CD28 stimulation is dependent on other cell types in PBMC. To investigate such possible stimuli for NK cells, for T cells, and for the other GM-CSF-producing cell types that are responsible for the above findings, we measured in in vitro stimulated PBMC supernatants the concentrations of the relevant cytokines that may regulate GM-CSF expression [11,12,17,20,22,28,29,30,31,32,33].

### 3.5. Supernatants of In Vitro Stimulated PBMC from MS Patients Contain Higher Levels of IL-2, IL-12, IL-1β, and GM-CSF and Lower TGF-β Levels than Those from Healthy Controls

**Figure 6:**
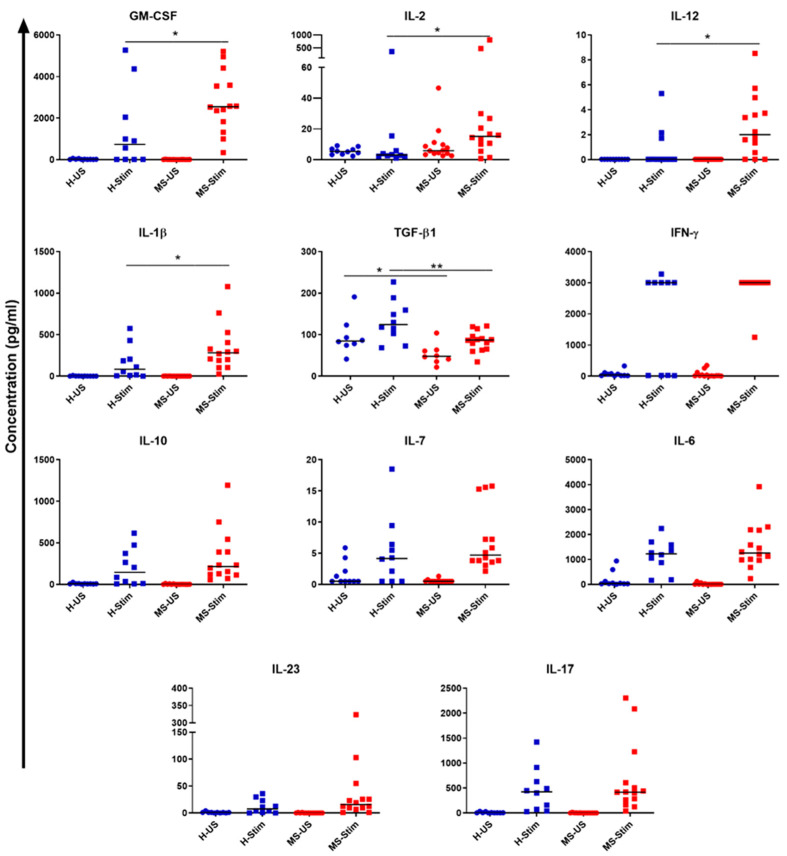
Cytokine profile in PBMC culture supernatants: Fresh isolated PBMC were cultured following anti-CD3/anti-CD28 protocol for 5 days. Culture supernatants were taken on day 5 from healthy controls (*n* = 10) and MS patients (*n* = 14), and profiles of cytokines both in unstimulated and stimulated conditions were determined as explained in the Subjects and Methods section. H-US and MS-US: unstimulated samples from healthy controls (H) and MS patients, respectively. H-Stim and MS-Stim: anti-CD3/anti-CD28-stimulated samples from healthy controls and MS patients, respectively. * *p* < 0.05 and ** *p* < 0.01. Horizontal lines are medians.

Supernatants collected from cultured and stimulated PBMC were examined to determine the levels of the following cytokines: GM-CSF, IFN-γ, IL-2, IL-7, IL-10, IL-12p70, IL-17, IL-23, IL-1β, IL-6, IL-15, IL-18, and TGF-β1. Among those, we found a statistically significant increase in the levels of GM-CSF, IL-2, IL-12, and IL-1β in MS patients when compared to HCs (Mann–Whitney, no correction for multiple measurements as nonindependent, HC *n* = 10; MS *n* = 14: GM-CSF *p* = 0.026; IL-2 *p* = 0.041; IL-12 *p* = 0.028; and IL-1β *p* = 0.041; Figure 6). On the other hand, we found a statistically significant decrease in TGF-β1 levels in MS patients when compared to HCs (unpaired *t*-test *p* = 0.0043, Figure 6). Higher pro-inflammatory cytokine concentrations accompanied by lower levels of the anti-inflammatory cytokine TGF-β may explain the dysregulation of GM-CSF in MS. This also suggests that one or more of the above inflammatory cytokines are responsible for the increased expression of GM-CSF (and possibly other inflammatory cytokines) in the PBMC from MS patients. Therefore, we performed experiments to block one or more of the cytokines which we found to be increased in MS PBMC supernatants (blocking IL-2, IL-12, IL-2 + IL-12, and IL-1β), and observed their effects on GM-CSF expression by Th cells, Tc cells, NK cells, and B cells. IL-15 and IL-18 levels were measured but were undetectable in the samples.

### 3.6. Blocking IL-2 In Vitro Reduced the Percentage of Th-GM Cells in Stimulated PBMC from MS Patients

**Figure 7:**
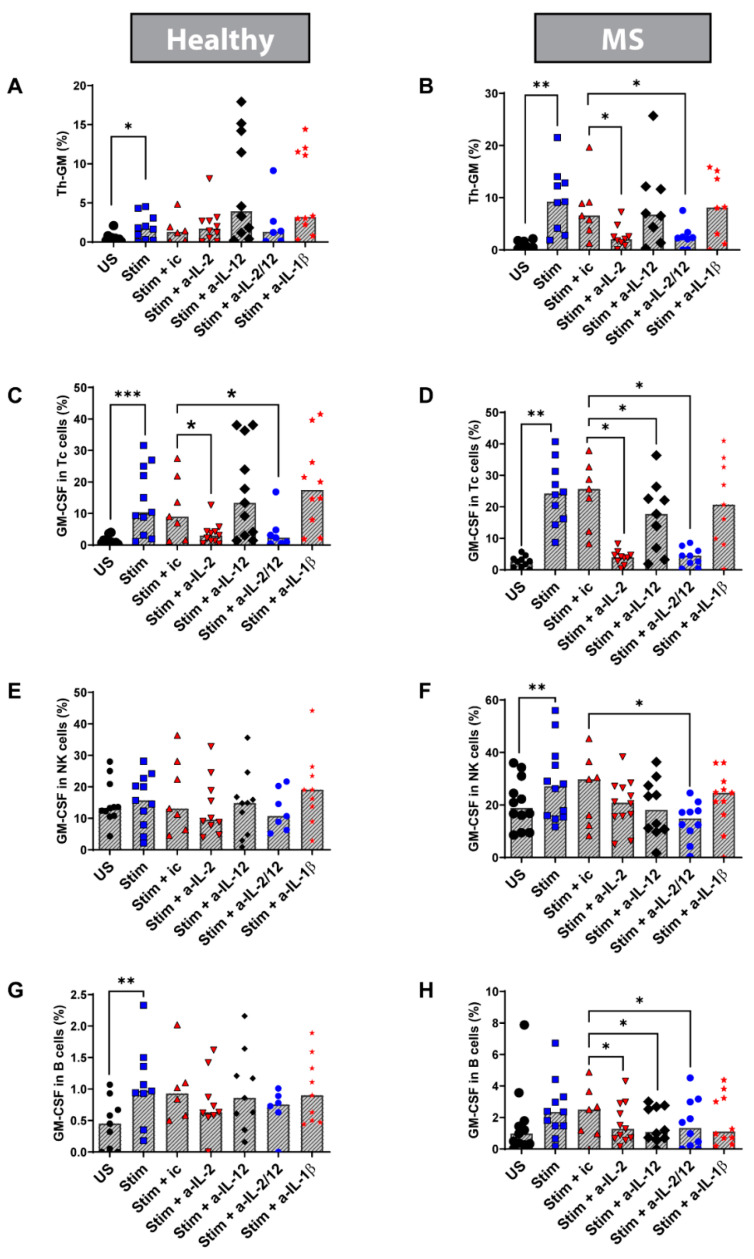
Effects of anti-cytokine antibodies on the frequency of GM-CSF-expressing PBMC subsets: Frozen/thawed cultured PBMC from healthy controls and MS patients were cultured either unstimulated (US) or stimulated (Stim) with anti-CD3/anti-CD28 for 5 days. Stimulated cells were left with or without adding one of the antibodies to block the following cytokine (a-cytokine: cytokine antibody): IL-2, IL-12, IL-2 + IL-12 (IL-2/IL-12), and IL-1β. In addition, stimulated cells were cultured with an antibody isotype control (ic) as the controls. The left panel represents healthy control results, and the right panel represents MS patient results. (**A**,**B**) Th-GM percentage; (**C**,**D**) percentage of cells expressing GM-CSF in Tc cells; (**E**,**F**) percentage of cells expressing GM-CSF in NK cells; (**G**,**H**) percentage of cells expressing GM-CSF in B cells. * *p* < 0.05 ** *p* < 0.01; and *** *p* < 0.001. Bars represent medians.

PBMCs were stimulated with anti-CD3/anti-CD28 in the presence of neutralizing antibodies to the above cytokines or isotype control antibodies. We found that blocking IL-2 (and IL-2 + IL-12) significantly reduced the frequency of Th cells expressing GM-CSF but not co-expressing IFN-γ or IL-17 (called Th-GM) in PBMC from MS patients but not in HCs (blocking IL-2: MS *n* = 9, Wilcoxon test *p* = 0.0039; HC *n* = 10, Wilcoxon test *p* > 0.99; Figure 7A,B). Blocking only IL-12 did not have any significant effects on these cells in PBMC. Blocking IL-2 + IL-12 had the same effect on Th-GM from MS patients but not on HCs, suggesting that IL-2 plays a key role in these effects (MS *n* = 8, Wilcoxon test *p* = 0.015; HC = 6, Wilcoxon test *p* = 0.43; Figure 7A,B). This indicates that IL-2 is an essential stimulus for this recently identified Th cell subset in MS. Specificity of this effect was verified by using an isotype control antibody. The overall Th1 and Th17 cells but not GM-CSF co-expressing Th1 and Th17 cells were also suppressed by the anti-IL-12 and by the anti-IL-2 plus anti-IL-12 antibodies in PBMC from both HC and MS patients (Figure S6). Altogether, these data indicate dysregulation of pro-inflammatory cytokines in MS and suggests reduced TGF-β in MS as a potential common mechanism.

### 3.7. Blocking IL-2 and IL-12 In Vitro Reduces the Frequency of Tc, NK, and B Cells Expressing GM-CSF in Stimulated PBMC from MS Patients

We examined the percentage of Tc cells, NK cells, and B cells expressing GM-CSF in cultured and stimulated PBMC. We found that blocking IL-12 significantly reduced the frequency of Tc cells expressing GM-CSF in MS (Wilcoxon test: MS *n* = 7, *p* = 0.046; HC *n* = 7, *p* = 0.57; Figure 7C,D). In addition, blocking IL-2 + IL-12 significantly reduced the percentage of NK cells expressing GM-CSF in MS (Wilcoxon test: MS *n* = 10, *p* = 0.01; HC *n* = 7, *p* = 0.53; Figure 7E,F). Moreover, we found that blocking either IL-2, IL-12, or both together significantly reduced the percentage of B cells expressing GM-CSF in MS but not in HC (Wilcoxon test, MS *n* = 7, blocking IL-2 *p* = 0.031; blocking IL-12 *p* = 0.046, blocking IL-2 + IL-12 *p* = 0.031; Wilcoxon test, HC *n* = 6, blocking IL-2 *p* = 0.06; blocking IL-12 *p* = 0.56; blocking IL-2 + IL-12 *p* = 0.093; Figure 7G,H). These results suggest dysregulation of GM-CSF across several immune cell types.

### 3.8. Th-GM Cells Differentiated In Vitro from Naïve CD4 T Cells Are More Frequent in Samples from MS Patients, and Exogenous IL-2 Increases Their Proportion Further

**Figure 8:**
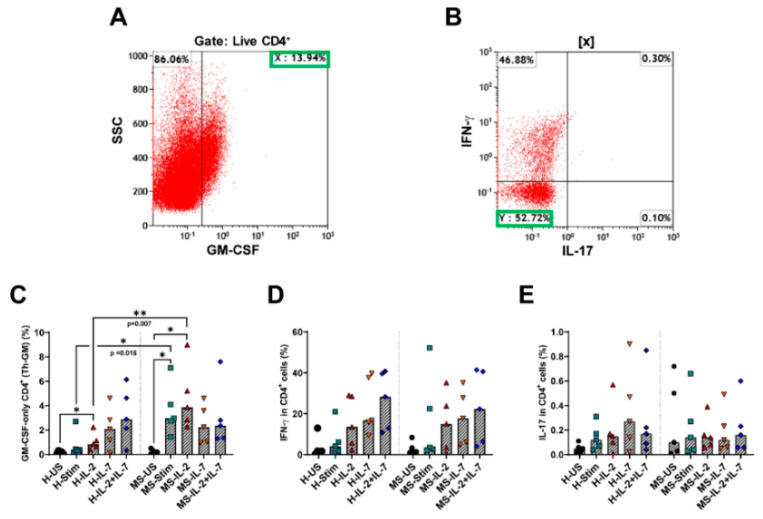
Frequency of cytokine-producing CD4^+^ T cells differentiated from naïve CD4 T cells in MS patient samples. Representative plots are shown in this figure. Freshly isolated (≥90% pure) naïve CD4 T cells (CD4^+^ CD45RA^+^) from healthy controls (H) and MS patients (MS) were left either unstimulated (US) or stimulated (Stim) with anti-CD3/anti-CD28 alone or with IL-2 and/or IL-7 for 7 days. (**A**) Identification of GM-CSF-expressing naïve CD4 cells (labelled as population X). (**B**) Using gating on population X, GM-CSF-only expressing cells were identified (labelled as population Y). The frequency of Th-GM cells among CD4 T cells was calculated according to the equation Th-GM (%) = X × Y × 100% (where X and Y are expressed as decimal values). (**C**) cumulative data from healthy controls and MS patients showing the frequency of Th-GM cells among CD4 T cells. (**D**,**E**) cumulative data from healthy controls and MS patients showing the frequency of IFN-γ^−^ and IL-17-producing CD4 T cells, respectively. Bars are medians. * *p* < 0.05 and ** *p* < 0.01.

It was shown that IL-2 and IL-7 may play a role in the differentiation of Th-GM cells from naïve CD4 T cells [17,20]. We aimed to determine whether the same pathways govern naïve CD4 T cell differentiation into Th-GM cells in humans and to investigate the frequency of these cells in MS patients compared to HC. Figure S7 shows the gating strategy for the analysis of these cells. We found that some naïve CD4 T cells differentiate into Th-GM after 7 days of stimulation with anti-CD3/anti-CD28. The frequency of Th-GM cells was higher in MS patients than in HCs (Mann–Whitney test *p* = 0.015; Figure 8C). When IL-2 was added to the medium, a higher proportion of Th-GM cells was observed in MS patients (Mann–Whitney *p* = 0.007), while IL-7 and the combination of IL-2 and IL-7 did not further affect the frequency of these cells. There was no detectible IL-3 expression after 7 days of culturing naïve CD4 T cells (results not shown). These findings complement the above results in PBMC, where neutralizing endogenously IL-2 in vitro reduced the frequency of Th-GM cells in MS patients but not in HCs. In these experiments, the above cytokines did not show differential effects on the expression of IFN-γ between MS and HC (Figure 8D,E, respectively).

## 4. Discussion

In MS, multiple neuroantigens are involved and the pathogenesis is not fully understood [34,35]. However, the common pathological process is the activation of inflammatory pathways, which involves antigen-specific T cells that stimulate more general immune cell activation and inflammation. Here, immune cells were stimulated to mimic antigen-specific activation and T cell receptor engagement plus co-stimulation, using antibodies against CD3 and CD28. Our aim was to determine if T cell-specific stimulation upregulates GM-CSF in T cells and in other immune cell types in the mixed PBMC; to investigate whether this upregulation is higher in MS; to identify factors (perhaps in addition to the known role of IL-2) that may be involved in this upregulation; and to determine whether immunoregulatory factors like TGF-β are produced in lower amounts in MS under these conditions.

To test the hypothesis that imbalance between pro-and anti-inflammatory factors, here focusing on GM-CSF in MS, is T cell driven, the CD3/anti-CD28 stimulation is relevant. It mimics antigen-specific activation of T cells [36,37]. This stimulation induced substantial and consistent cytokine production in PBMC; similar protocols were previously used by our groups and others in studies of this subject [19,22]. We found that activated Th1 and Th17 cells express GM-CSF and that the frequency of GM-CSF-expressing Th1 cells is higher in PBMC from MS patients than HC. This is associated with higher levels of Th1 inducing IL-12 in PBMC from MS patients. Higher IL-2 levels detected in this work and the association of IL-2R polymorphisms reported in MS with higher production of GM-CSF by T cells [22] may contribute to this finding. We also detected GM-CSF-only expressing Th cells and found their frequency to be higher in MS. These cells (called Th-GM cells) being increased in MS are likely to have a pathogenic role, supported by their expansion in the CNS in MS together with CXCR4 [24]. These cells also express integrin α4β1 (very late antigen-4, VLA-4) and probably have different migration properties than the other Th subsets [20,21,22,23,24]. Of note, cells named here Th-GM (for simplicity) were shown in this study to express GM-CSF but not IFN-γ, IL-17, or IL-3 (to compare them to the murine counterpart described by Sheng and colleagues [17]); we have not tested the coexpression of other cytokines that can coexist with GM-CSF [24] such as IL-9, IL-22, IL-21, etc.

We also show that GM-CSF is expressed at higher frequency by in vitro stimulated Th and Tc cells in MS patients when compared to HC. This is consistent with recent studies [19,22] and indicates that GM-CSF has a crucial role in autoimmune inflammation. We found that GM-CSF is more highly expressed by monocytes from MS patients in mixed PBMC upon T cell stimulation.

Our results also demonstrate a higher proportion of GM-CSF-expressing B cells in PBMC from MS patients after in vitro stimulation. This is consistent with the findings of a seminal study performed on B cells after their isolation and stimulation with either PMA/I or CD40L^+^ anti-IgM [38]. Our results suggest that T cells can stimulate B cell GM-CSF production.

We also show a higher frequency of NK cells expressing GM-CSF in PBMC from MS patients when compared to healthy controls after in vitro T-cell dependent stimulation. A previous study has found an increase in NK cells in PBMC during the remission stage of MS. These cells were of the NK2 subtype exerting anti-inflammatory actions including the suppression of Th1 responses [39]. It has been reported that peripheral NK2 and NK1 cells produce similar amounts of GM-CSF [40]. The elevation of the percentage of GM-CSF-expressing NK cells in MS may indicate a potential pathogenic role.

Indirect stimulation of NK cells with a T cell stimulus (anti-CD3/anti-CD28) induced a higher frequency of GM-CSF-expressing NK cells in MS. Here, investigating GM-CSF expression in isolated NK cells using stimuli known to induce high GM-CSF concentrations in NK cells [28], we show similar GM-CSF expression in MS patients and HC. This indicates that the factors that are more abundant in MS and that induce GM-CSF in MS NK cells are dependent on T cell activation.

In this study, the levels of GM-CSF, IL-2, IL-12, and IL-1β were elevated, and the levels of TGF-β1 were reduced in the culture supernatants of in vitro stimulated PBMC from MS patients.

Our study confirms the role of IL-2 in the upregulation of GM-CSF in MS already shown in a previous study [22]. Complementing that study where exogenous IL-2 stimulated Th expression of GM-CSF to higher levels in MS, here, we show that anti-IL-2 suppresses it and not only in Th cells but also other cell types. Although that study did not identify the Th subsets with increased GM-CSF production, it found that GM-CSF and IFN-γ expression (but not IL-17) was increased in MS, raising the possibility that these GM-CSF-expressing cells are Th1 cells and the newly identified Th cell subset (Th-GM cells) but not Th17 cells. Subsequent studies by the same group using single cell sequencing identified those MS-relevant cells as pathogenic CXCR4^+^ Th cells. Our results are also consistent with the findings of a recent study showing that GM-CSF but not IFN-γ was induced by IL-2 to be expressed by Th cells. These cells were distinct from the classical Th1 or Th17 subsets and had different migration properties [20,23]. We hereby confirm and further describe the GM-CSF-non-Th1-nonTh17-producing Th cell subset that is increased in MS; IL-2 potentially plays an important role in their differentiation.

We also found that blocking IL-12 significantly reduced the frequency of Tc cells expressing GM-CSF in MS patients but not in healthy controls. IL-12 is involved in the generation of Tc cells [33]. It synergizes with IL-2 to increase GM-CSF expression [33]. It was shown that IL-12A and IL-12B genetic polymorphisms are genetic risk factors for MS [32], which may lead to increased IL-12-induced GM-CSF expression.

The results of this work show a reduction in the frequency of CD3^-^CD56^+^ PBMC (NK cells) expressing GM-CSF after blocking both IL-2 and IL-12 in PBMC from MS patients after anti-CD3/anti-CD28 stimulation but not in those from healthy controls. These results complement previous reports that IL-2 and IL-12 induce NK cell cytotoxicity and GM-CSF production [41,42,43,44]. The differential effects between stimulated cells from MS and controls may again be attributed to the known MS-associated polymorphisms in the IL-12 subunits, IL-12R, and IL-2R genes, as previously demonstrated for T cell-derived GM-CSF [22,32].

Here, the blockade of IL-2 significantly reduced the percentage of GM-CSF-expressing B cells in stimulated PBMC from MS patients but not healthy controls. IL-2R-α (CD25) is expressed on immature [45] B cells, and IL-2 induces B cell proliferation and differentiation into plasma cells [46,47,48,49]. This is probably the first report of the relation between IL-2 and GM-CSF expression in B cells. On the other hand, as cytokine expression may reflect lymphocyte activation [50], reduced activation and proliferation of B cells by blocking IL-2 may have resulted in reduced GM-CSF expression. This effect was statistically significant in MS patients, possibly because of higher IL-2 levels in MS patients, or because the same MS-associated IL-2R polymorphism governs GM-CSF production by B cells as in T cells. GM-CSF may exert an autocrine effect increasing B cell survival [51], which may be pathogenic.

We show that blocking IL-12 reduces GM-CSF expression by B cells. While IL-12 induces GM-CSF expression in T cells [52], this was not explored thoroughly in B cells. A previous study has shown IL-12 to induce B cell differentiation into a distinct subset similar to Th1 cells [53]. We found significantly higher IL-12 levels in supernatants of stimulated PBMC from MS patients compared to HCs and MS patients. Our results show for the first time that IL-12 is nonredundant for GM-CSF expression by B cells in mixed immune cell populations stimulated with a T cell stimulus in MS patients compared to HC. This may be explained by concentration-related effects of IL-12 on GM-CSF expression by B cells in MS or by polymorphisms in IL-12 or IL-12R, which are genetic risk factors of MS [54,55].

The upregulation of GM-CSF across cell lineages may reflect a higher state of activation of immune cells from people with MS compared to healthy controls. Our previous studies have shown increased activation markers such as CD69 and CD154 on T cells from MS patients compared to healthy controls, either ex vivo without stimulation or after in vitro stimulation as in the current paper [56]. This likely reflects genetically determined enhanced IL-2 responsiveness in MS. In view of this, we have not corrected our intracellular cytokine staining findings by activation marker expression, in particular since basal unstimulated production of GM-CSF usually tends to be minimal. Although attributing the upregulation of GM-CSF merely to higher T cell activation in MS does not reduce the significance of the finding, we postulate a more specific effect. First, the same upregulation was not seen for other proinflammatory cytokines in this study. Secondly, other activating stimuli do not differentially induce GM-CSF in cells from people with MS or healthy control volunteers. Third, both the enhanced activation of GM-CSF across immune cell types and the higher immune activation state may result from immune dysregulation, e.g., reduced TGF-β. The reduction in TGF-β is a key finding of our study.

TGF-β is an anti-inflammatory cytokine that inhibits the differentiation, proliferation, and functioning of Th1 and Th2 cells. However, it is involved in Th17 differentiation in the presence of IL-6. It is also involved in the differentiation of Treg in the presence of IL-2. TGF-β also supresses NK and B cell functioning and maturation [57,58]. Our finding of lower TGF-β levels in PBMC culture supernatants of MS patients is relevant and provides a plausible mechanism for the upregulation of GM-CSF across immune cell lineages along with elevated pro-inflammatory cytokines in MS. Low TGF-β may result in numerical or functional decreases of Treg in MS patients, which inhibit GM-CSF expression by immune cells. Other studies have shown contradicting findings in terms of serum levels of TGF-β in MS, being elevated in some [59] and reduced in others [60]. Our study is different in that it tests culture supernatants but not serum levels.

We also examined whether the Th-GM cells differentiated from naïve CD4 T cells as identified in mice [17] are also present in humans and whether there are differences between MS and HC. Therefore, IL-7 was used for differentiation and IL-2 was used alone or with IL-7. Of note, adding IL-2 (10 ng/mL) to CD4 memory T cells for 5 days significantly increases GM-CSF-producing Th cells without affecting IFN-γ or IL-17 expression [20]. Our results suggest that anti-CD3/anti-CD28-stimulated naïve CD4 T cells probably differentiate to Th-GM cells. The frequency of Th-GM cells was higher in stimulated Th cells from MS patients than HC. IL-2 increased the frequency of Th-GM in cultured Th cells from MS patients. These results add to the evidence for the role of IL-2 in GM-CSF production and its increase in MS.

## 5. Conclusions

In conclusion, multiple immune cell types in a mixture that reflects peripheral blood composition show increased pro-inflammatory cytokine GM-CSF production upon in vitro stimulation with T cell stimuli in PBMC from patients with MS. This may have wider implications, as it is likely that a similar dysregulation is present in other inflammatory autoimmune diseases, where GM-CSF has been implicated [61]. The addition of a positive control group with other inflammatory diseases in parallel to the present MS group would have strengthened our results. From a therapeutic perspective, it was the promising results of GM-CSF blockade in rheumatoid arthritis (reviewed in [62]) that encouraged investigation of a similar approach in MS [63]. For example, a human monoclonal antibody was shown to be safe and well tolerated in phase I clinical trials in MS and rheumatoid arthritis [64,65,66]. Thus, targeting dysregulated GM-CSF or its receptor is a potentially effective and safe addition to the therapeutic armamentarium in MS and possibly other autoimmune diseases.

## Supplementary Materials

Supplementary Materials are available online at https://www.mdpi.com/2227-9059/8/7/226/s1.
