# Supplementary material for: Increased IL-2 and Reduced TGF-β Upon T-Cell Stimulation are Associated with GM-CSF Upregulation in Multiple Immune Cell Types in Multiple Sclerosis
Source: Biomedicines. 2020 Jul 18;8(7):226. doi: 10.3390/biomedicines8070226 (PMC7400438; doi:10.3390/biomedicines8070226)
Supplement: Supplementary file 1 [file biomedicines-08-00226-s001.pdf]

biomedicines

Supplementary figures for manuscript: "Increased IL-2 and reduced TGF-β upon T-cell stimulation are associated with of GM-CSF Up-regulation in Multiple Immune Cell Types in Multiple Sclerosis" by Jehan Aram et al.

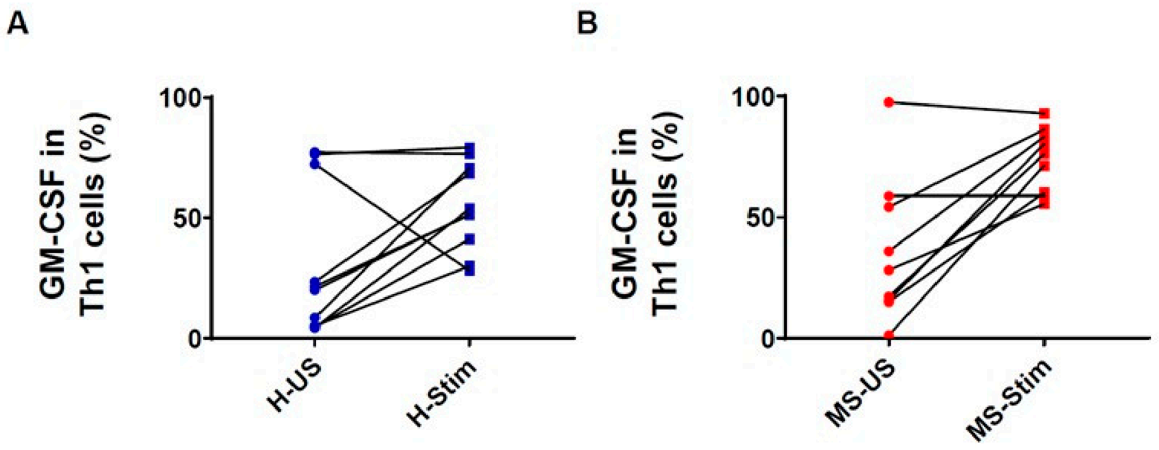

Figure S1. GM-CSF expression inTh1 cells. Figure shows the paired US/S data for each healthy control and MS sample. (**A**)Percentage of Th1 GM-CSF producing cells in healthy control, before and after stimulation. (**B**) Percentage of Th1 GM-CSF producing cells in MS before and after stimulation.

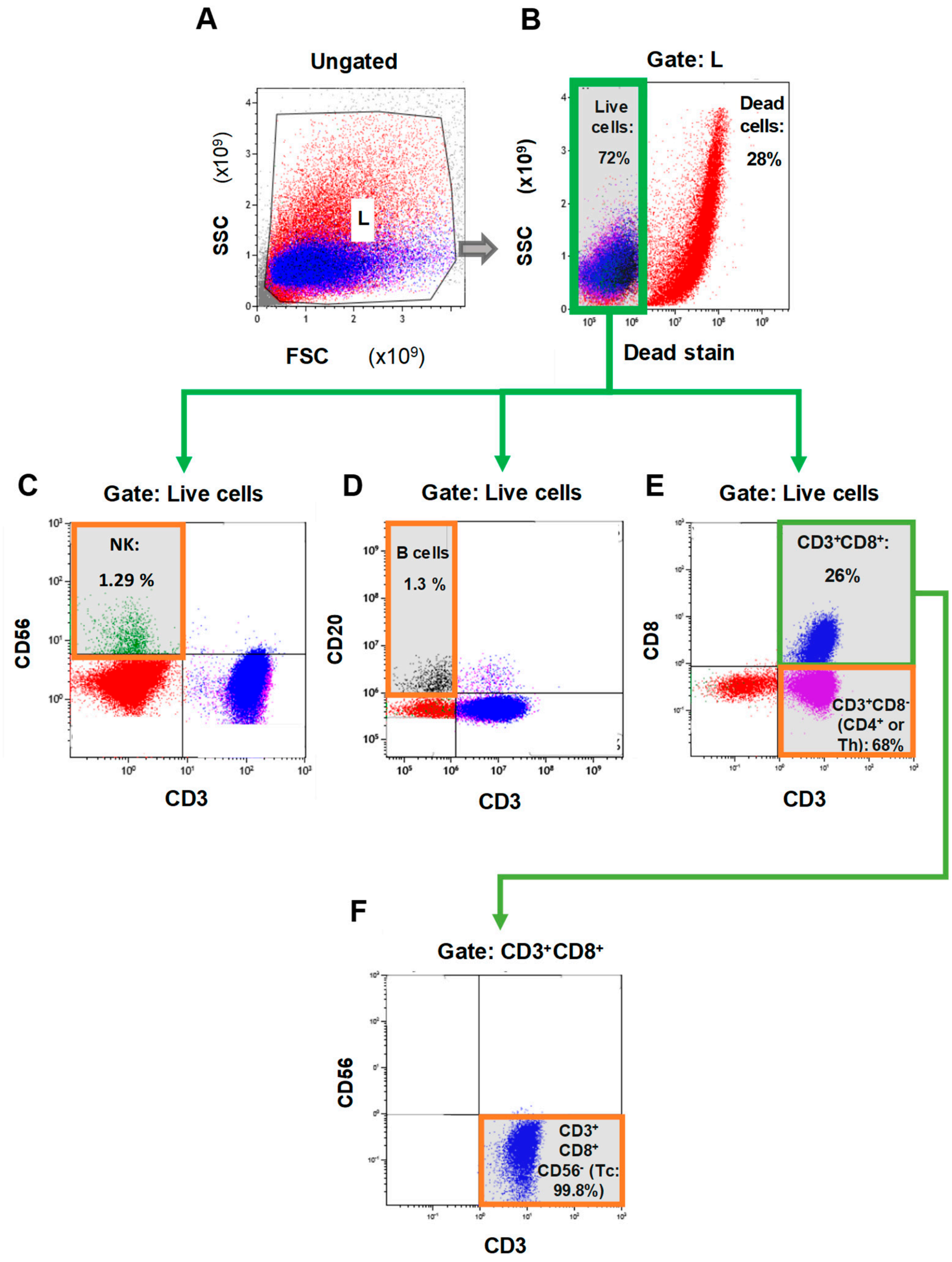

Figure S2. Cell gating, selection and identification in flow cytometry. (**A**) selecting potential cells (L); **B:** selecting live cells and using it as a gate for later plots; (**C**) NK cells were identified as CD3-CD56$^+$ cells (green-coloured population); (**D**) B cells were identified as CD3$^-$CD20$^+$ cells (black-coloured population); (**E**) Th cells were identified as CD3$^+$CD8$^-$ cells (purple-coloured population); CD3$^+$CD8$^+$ gate was use in (**F**) Tc cells were identified as CD3$^+$CD8$^+$CD56$^-$ cells (blue-coloured population). Representative plots are shown.

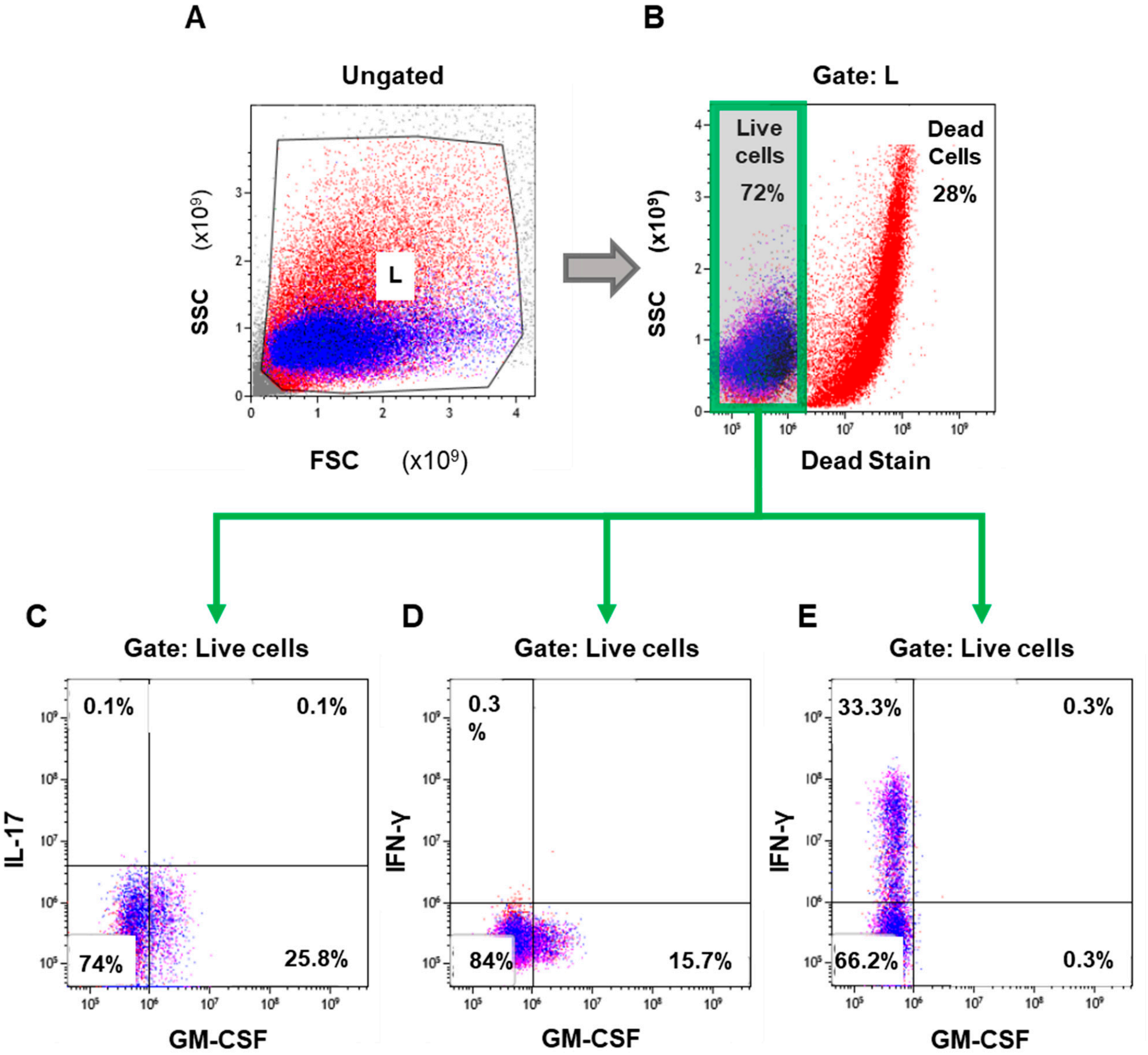

Figure S3. Setting flow cytometry quadrants using fluorescence minus one (FMO) controls. (**A**) selecting potential cells (L); (**B**) selecting live cells and using it as a gate for later plots; (**C–E**) represenatative FMO control plots for IL-17, IFN-γ, and GM-CSF, respectively.

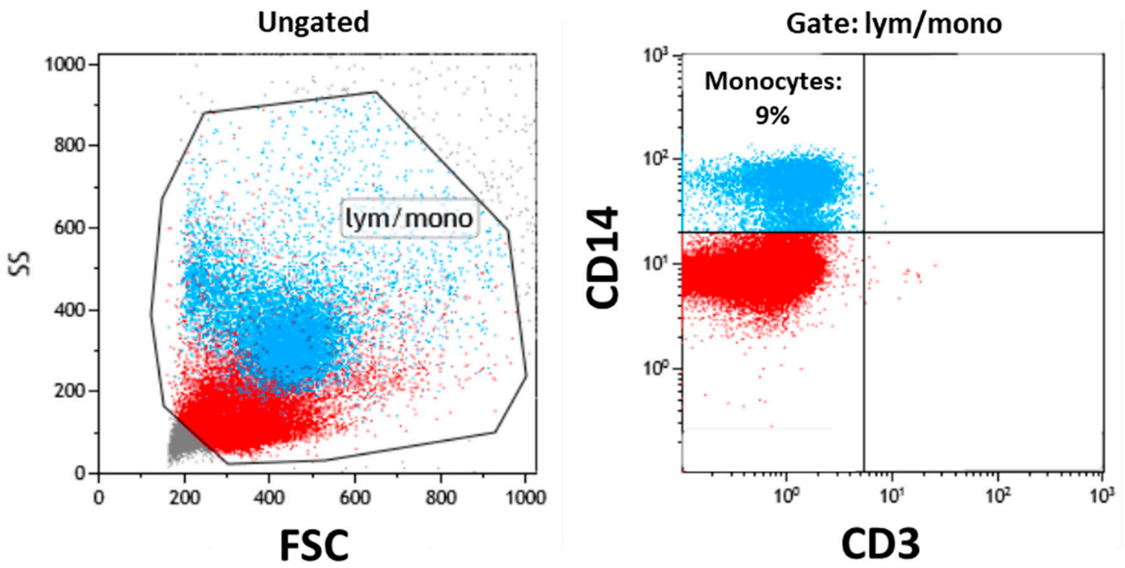

Figure S4. Selection and gating for monocytes. After selection of potential cells (lym/mono gate), monocytes were identified as CD3⁻CD14⁺ cells (light blue colored population).

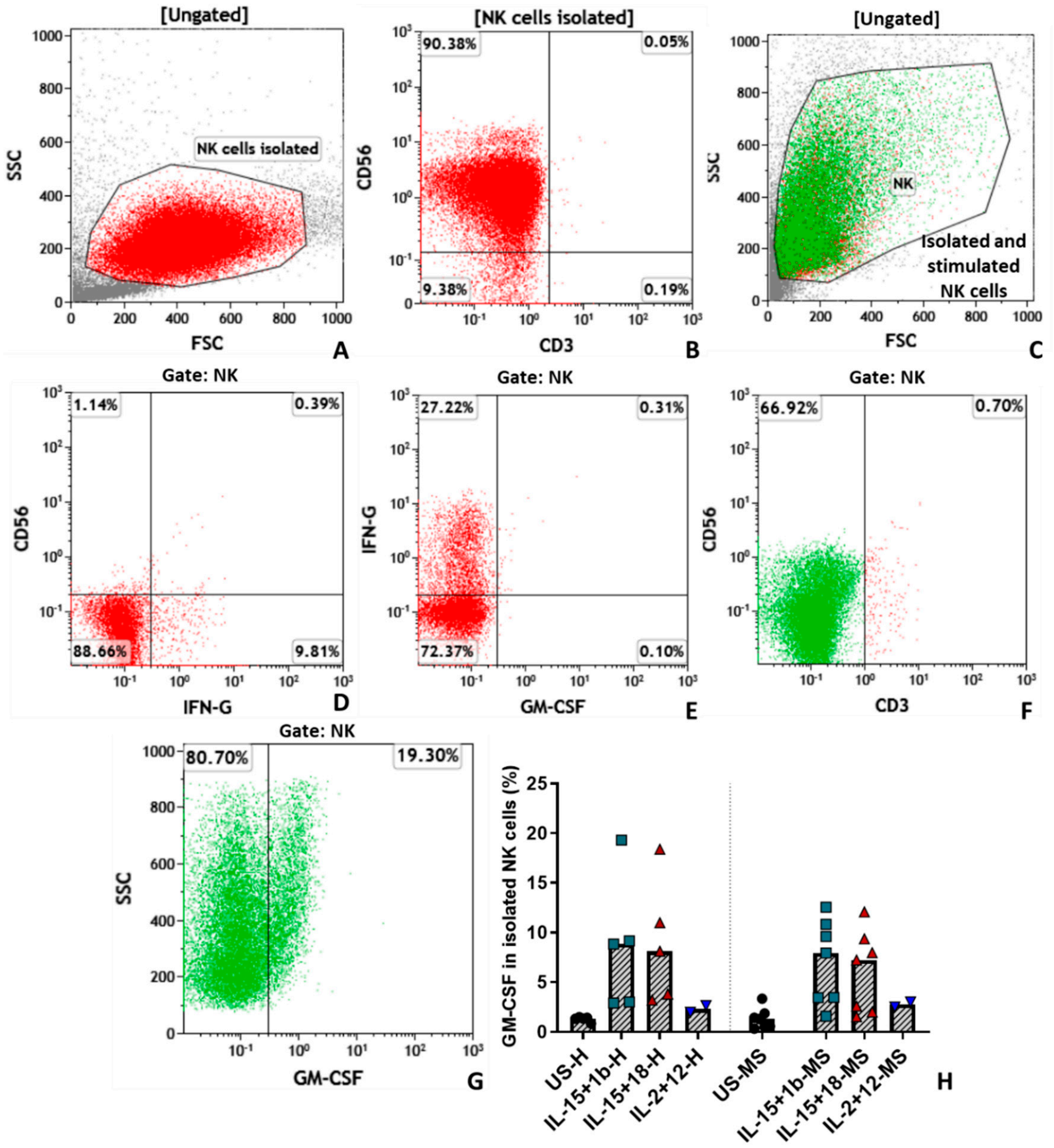

Figure S5. GM-CSF expression in isolated NK cells. After magnetic isolation of NK cells from freshly isolated PBMC, they were checked for purity. (**A**) forward and side scatter (FSC and SSC) plot for the freshly isolated NK cells. (**B**) purity check of freshly isolated NK cells gated by the cells selected in plot A, showing ≥ 90% $CD3^-CD56^+$ cells. After purity check, NK cells were either left unstimulated (US) or stimulated with either IL-15+IL-1β, IL-15+IL-18, or IL-2+IL-12 for 3 days, then re-stimulated for the last 5 hrs. with PMA/I in presence of brefeldin A. (**C**) the FSC and SSC plot to gate the possible NK cells after stimulation. (**D**) FMO control of CD56, (**E**) FMO control of GM-CSF. (**F**) gating cells to select $CD3^-$ NK cells (green-colored population) (note that CD56 expression was down-regulated in purity-validated NK cells after being cultured and stimulated). (**G**) GM-CSF expression by NK cells. (**H**) collective results from healthy controls (**H**) and MS patients illustrating the frequency of GM-CSF-expressing NK cells ($GM\text{-}CSF^+$ cells in plot (**G**), horizontal lines are medians. Each experiment was independently performed for at least three times. Plots from A-G are representative and could be taken from different samples and are not necessarily from the same sample.

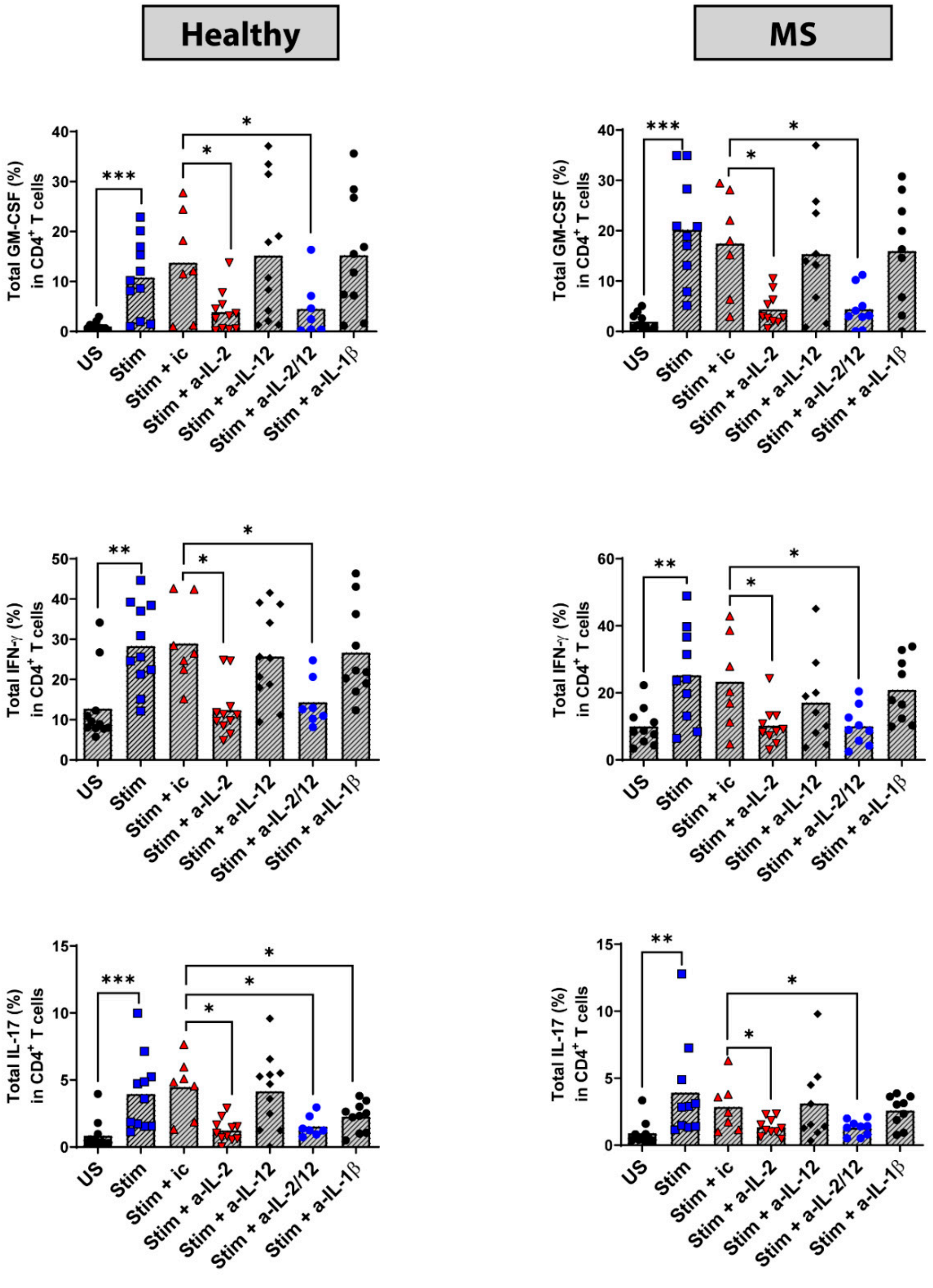

Figure S6. Effects of anti-cytokine antibodies on CD4+ T cells expressing either GM-CSF, IFN- or IL-17. Frozen/thawed cultured PBMC from healthy controls and MS patients were cultured either unstimulated (US) or stimulated (Stim) with anti-CD3/anti-CD28 for 5 days. Stimulated cells were left with or without adding one of the antibodies to block the following cytokine (a-cytokine: cytokine antibody): IL-2, IL-12, IL-2 + IL-12 (IL-2/IL-12) and IL-1β. In addition, stimulated cells were cultured with antibody isotype control (ic) as controls. Left panel represents healthy control results, right panel represents MS patient results * $p<0.05$ ** $p < 0.01$; *** $p < 0.001$. Bars represent medians.

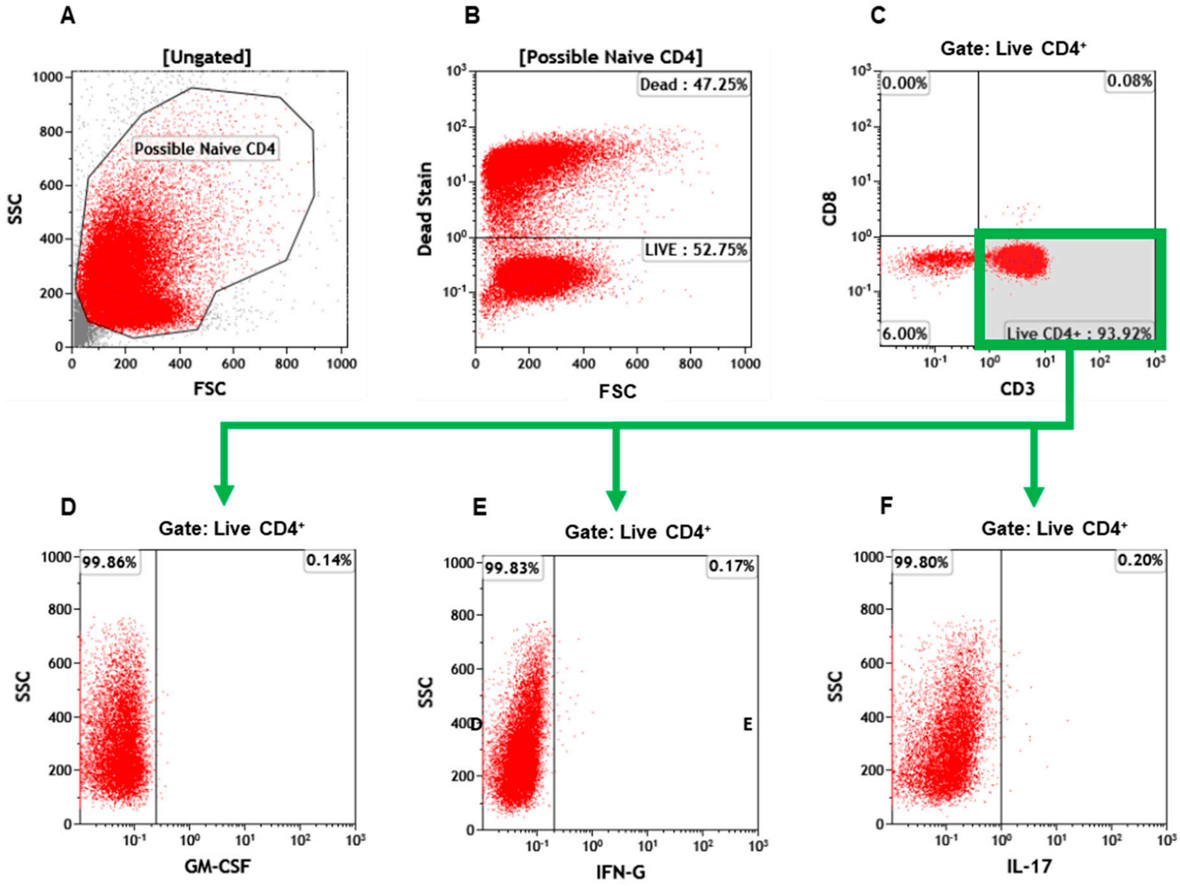

Figure S7. Naïve CD4 cell gating, selection, and FMO controls. After magnetic isolation of naie CD4 cells from freshly isolated PBMC, they were checked for purity (≥ 90% naïve CD4 T cells ($CD4^+CD45RA^+$) were included). (**A**) FSC and SSC plots to select possible Naïve CD4 T cells. (**B**) from the cells selected in A, live cells were identified and selected as a gate for the next plot. (**C**) Live $CD4^+$ ($CD3^+CD8^-$) T cells were identified and selected as a gate for the following plots. (**D–F**): fluorescence minus one (FMO) controls for GM-CSF, IFN-γ, and IL-17, respectively. They were used to set the quadrants for the next plots. Representative plots were shown in this figure.

Table S1. Demographic and clinical characteristics of the patients.

ID #	Gender	Age (years)	MS type	EDSS	Prior DMT	Reason off	MS duration (Years)	Exp. Figure
**1**	M	36	RR	5	IFN	Switch	2	5
**2**	M	34	RR	6	IFN	Switch	2	5
**3**	F	33	RR	6	Nil	Pt choice	10	5
**4**	F	30	RR	2	Nil	Early MS	1	5
**5**	F	67	SP	6.5	Nil	SPMS	15	5

**6**	F	45	RR	2	Nil	Pt choice	7	5
**7**	F	32	RR	4	Nil	Early MS	2	5
**8**	F	67	RR	1	Nil	Benign MS	16	5
**9**	F	49	RR	1.5	Nil	Pt choice	14	5
**10**	M	58	SP	6	Nil	Side effects	6	5
**11**	F	53	RR	2	Nil	Benign MS	8	5
**12**	F	50	SP	6.5	Nil	SPMS	14	5
**13**	M	40	SP	5	Nil	SPMS	10	5
**14**	M	64	SP	5	Nil	SPMS	3	5
**15**	M	50	SP	6.5	Nil	SPMS	12	5
**16**	F	39	RR	1	Nil	Early MS	<1	5
**17**	F	49	SP	6	IFN	Stop	4	5
**18**	F	33	RR	2	Daclizumab	Stop	4	2,3,6
**19**	M	43	SP	2	GA	SPMS	6	2,3,6
**20**	M	43	RR	2	Nil	Pt choice	5	2,3,6
**21**	F	33	RR	3	GA	Switch	7	2,3,6
**22**	F	38	RR	3	Nil	Pt choice	8	2,3,6,8
**23**	F	56	RR	3.5	IFN	Switch	7	2,3,6
**24**	F	49	RR	4	IFN	Switch	27	2,3
**25**	F	51	SP	6	Nil	SPMS	11	2,3
**26**	F	38	RR	3	Nil	Early MS	1	2,3,6
**27**	M	18	RR	2.5	Nil	Early MS	2	S5, 6
**28**	F	52	RR	3	Nil	Pt choice	7	S5, 6
**28**	F	43	RR	2	Nil	Pt choice	9	S5, 6
**30**	M	51	SP	5.5	Azathioprine	Stop	11	S5, 6
**31**	F	58	RR	6	IFN	Stop	10	S5, 6
**32**	F	31	RR	3	IFN	Stop	6	S5, 6
**33**	F	41	RR	2	GA	Pt choice	14	S5, 6
**34**	F	34	RR	2	GA	Switch	4	1,4,7
**35**	F	27	RR	1.5	IFN	Switch	2	1,4,7
**36**	F	23	RR	1	Nil	Early MS	2	1,4,7
**37**	F	55	RR	1.5	Nil	Benign MS	14	1,4,7
**38**	F	33	RR	2.5	Nil	Pt choice	1	1,4,7
**39**	F	42	RR	1	Nil	Pt choice	1	1,4,7
**40**	F	30	RR	2	Nil	Trial entry	10	1,4,7
**41**	F	34	RR	3	Fingolimod	Pregnancy	8	1,4,7
**42**	F	55	RR	1	Nil	Early MS	<1	1,4,7
**43**	M	57	RR	2	Nil	Not criteria	8	1,4,7
**44**	F	67	RR	5.5	Nil	Not criteria	4	8
**45**	M	21	RR	1	Nil	Early MS	<1	8

**46**	F	38	RR	5.5	GA	Side effects	10	8
**47**	F	42	RR	4	IFN	Switch	8	8

RR, relapsing remitting; SP, secondary progressive; EDSS, Expanded Disability Status Scale; DMT, disease modifying treatment; IFN, interferon beta; GA, Glatiramer acetate; Reason off: reason the patient was off DMT at the time of sample collection; Exp. Figure: sample contribution to the experiments shown in figure with the number given in the table.
